# 石墨烯应用于样品前处理的研究进展

**DOI:** 10.3724/SP.J.1123.2022.07012

**Published:** 2022-11-08

**Authors:** Juanjuan FENG, Mingxia SUN, Yang FENG, Xubo XIN, Yali DING, Min SUN

**Affiliations:** 济南大学化学化工学院, 山东 济南 250022; School of Chemistry and Chemical Engineering, University of Jinan, Jinan 250022, China

**Keywords:** 石墨烯, 氧化石墨烯, 样品前处理, 固相萃取, 固相微萃取, 萃取材料, 金属离子, 有机污染物, 综述, graphene, graphene oxide, sample preparation, solid-phase extraction, solid-phase microextraction, extraction materials, metal ions, environmental pollutants

## Abstract

样品前处理技术在样品分析中发挥着越来越重要的作用,而对分析物的富集能力和对样品基体的净化程度主要取决于高效的样品前处理材料,所以发展高性能的样品前处理材料一直是该领域的前沿研究方向。近年来,各类先进材料已经被引入样品前处理领域,发展了多种高性能的萃取材料。由于独特的物理化学性质,石墨烯已在各个研究领域获得广泛关注,在样品前处理领域也发挥着重要作用。基于高的比表面积、大的*π*电子结构、优异的吸附性能、丰富的官能团和易于化学改性等优点,石墨烯和氧化石墨烯基萃取材料被成功应用于各种样品的前处理,对不同领域中多种类型分析物表现出优异的萃取性能。该论文总结和讨论了近3年来石墨烯材料(石墨烯、氧化石墨烯及其功能化材料)在柱固相萃取、分散固相萃取、磁性固相萃取、搅拌棒萃取、纤维固相微萃取和管内固相微萃取等方面的研究进展。基于多种萃取机理如*π-π*、静电、疏水、亲水、氢键等相互作用,石墨烯萃取材料能够高效萃取和选择性富集不同类别的目标分析物,如重金属离子、多环芳烃、塑化剂、雌激素、药物分子、农药残留、兽药残留等。基于新型石墨烯萃取材料的各种样品前处理技术与多种检测技术如色谱、质谱、原子吸收光谱等联用,广泛应用于环境监测、食品安全和生化分析等领域。最后,总结了石墨烯在样品前处理领域中存在的问题,并展望了未来的发展趋势。

## Abstract (en)

Sample preparation is playing an increasingly important role in sample analysis. The enrichment efficiency of the target and the removal effect of the sample matrix are strongly dependent on the extraction material. Therefore, the development of efficient extraction materials is an important research focus in the field of sample preparation. Various advanced materials such as nanomaterials, mesoporous materials, ionic liquids, aerogels, carbon materials, metal-organic frameworks, and covalent organic frameworks have been introduced to produce a diverse range of extraction materials for sample preparation. Owing to its unique physical and chemical properties, graphene, an excellent carbon nanomaterial, has attracted significant attention in different areas. Due to their unique advantages of large surface area, large *π*-electrons, excellent adsorption properties, abundant functional groups, and facile chemical modification, graphene-based materials have displayed excellent extraction performance for diverse analytes. Furthermore, graphene-based extraction materials have been applied to pretreat real samples from different fields. This paper provides an overview of the recent advances in graphene sample preparation from 2020 to date. The manuscript covers the use of graphene, graphene oxide, and the related functionalized materials as sorbents, as well as their specific applications in cartridge solid-phase extraction, dispersive solid-phase extraction, magnetic solid-phase extraction, stir bar sorptive extraction, fiber solid-phase microextraction, and in-tube solid-phase microextraction. To prevent the aggregation of graphene, three-dimensional graphene, porous graphene aerogels, graphene-modified silica, and stainless-steel mesh were developed for cartridge solid-phase extraction. Furthermore, some graphene-based extraction materials were used to develop online solid-phase extraction, which allowed for automatic and high-throughput tests. Graphene nanosheets and their hybrid materials with molybdenum disulfide or zinc oxide nanoparticles have been applied to dispersive solid-phase extraction, and several types of contaminants, including metal ions, bisphenol endocrine disruptors, paraben preservatives, and phthalates, could be captured. By combination with magnetic materials using the coprecipitation method or via chemical post-modification, many magnetic graphene extraction materials have been produced for magnetic solid-phase extraction. The introduction of magnetic graphene not only enhanced the extraction efficiency but also simplified the test process, making it highly suitable for complex samples such as food and biological samples. Similar to magnetic solid-phase extraction, stir bar sorptive extraction is a very simple and efficient extraction method that shows good extraction performance for metal ions and organic pollutants from environmental water, medicines in urine, and organic pollutants in cosmetics. In addition to its excellent applicability to solid-phase extraction, graphene delivered satisfactory performance for solid-phase microextraction. Graphene has been used as an extraction coating for the extraction of fibers or tubes by coupling solid-phase microextraction with chromatographic detection, and many kinds of organic pollutants, including polychlorinated biphenyls, phthalates, polycyclic aromatic hydrocarbons, toluene, xylenes, organophosphorus pesticides, phenoxy acid herbicides, and antibiotics, in environmental or biological samples have been successfully determined. The extraction mechanism, including *π-π*, electrostatic, hydrophobic, hydrophilic, and hydrogen-bonding interactions, is also discussed. Because of the mixed-mode interactions and rich functionalization, graphene-based extraction materials could effectively capture and selectively enrich different types of species. These extraction or microextraction techniques have been coupled with detection methods such as chromatography, mass spectrometry, and atomic absorption spectroscopy and widely used in environmental monitoring, food safety, and biochemical analysis.

The future development of graphene in the field of sample pretreatment focuses on the following aspects: 1) functionalization of graphene with specific groups such as affinity groups, chelating groups, and molecularly imprinted sites to achieve unique extraction selectivity; 2) combination of graphene with the advanced materials, including covalent organic frameworks, metal organic frameworks, aerogels, and nanomaterials, thus realizing the complementary advantages between materials, so that the hybrid graphene materials find broad application prospects in sample preparation; 3) combination of electromagnetic materials with graphene to form electromagnetic composites, as well as the use of electromagnetic fields to improve extraction selectivity and efficiency; 4) exploiting the good performance of graphene-based materials to overcome the difficulty encountered in the pretreatment of complex samples; 5) development of more green methods to prepare graphene-based extraction materials or functionalize graphene, in line with the trends in green chemistry; 6) application of more graphene-based materials to online sample preparation for meeting the development trends in the field of analytical chemistry.

目前,人们在应对分析任务时要面临两个主要问题:一是样品基体复杂会严重干扰准确测定;二是目标分析物是痕量或超痕量水平,现有仪器和方法难以灵敏检测。为了达到准确且灵敏的分析,在进行仪器检测之前,样品前处理是必不可少的。近年来,固相萃取和固相微萃取等样品前处理技术已经吸引了越来越多的关注,并被广泛应用于环境、食品、生物、医药等诸多领域。各种类型的先进材料如介孔材料^[1]^、纳米材料^[2]^、离子液体^[3]^、气凝胶^[4]^、金属有机框架材料(MOFs)^[5]^等被应用于发展高效的萃取材料,促进了样品前处理技术的发展。

石墨烯是一种以*sp*^2^杂化碳原子相互连接、紧密堆积成单层二维蜂窝状晶格结构的新型碳纳米材料,具有优异的光学、电学、力学特性,以及超高的比表面积、强的疏水性和良好的化学稳定性。然而,石墨烯的强疏水性也导致其在水相中易团聚,导致高比表面积的优势得不到充分发挥。氧化石墨烯除了具有石墨烯的微观结构和化学组成外,还带有丰富的含氧官能团如羧基、羟基、醚基等,除了表现出良好的亲水性外,这些基团的存在也利于进一步化学修饰改性。石墨烯和氧化石墨烯已经被应用于各个领域,表现出优异的物理化学性能,并且被引入到样品前处理从而发展了多种高性能的前处理材料。本论文聚焦于2020年初至今石墨烯和氧化石墨烯在样品前处理领域的最新研究进展,重点综述了石墨烯、氧化石墨烯以及它们的功能化材料作为新型高效的萃取材料在柱固相萃取、分散固相萃取、磁性固相萃取、搅拌棒萃取、纤维固相微萃取、管内固相微萃取等多种样品前处理技术中的具体应用,以及成功适用于环境监测、食品安全和生化分析等领域。

## 1 柱固相萃取

柱固相萃取作为一种常用的样品前处理技术,其吸附剂为硅胶或硅胶基质的复合材料,为了克服硅胶吸附剂的缺点以及满足不同的需要,其他材料如离子液体、碳纳米管、石墨烯等也逐渐被应用于柱固相萃取。

石墨烯因具有大的比表面积、丰富的电子以及双面多苯环骨架而表现出优异的萃取性能,是一种有前途的吸附剂材料。Feizy等^[6]^制备了石墨烯纳米颗粒,并将其装填进固相萃取柱中,通过与高效液相色谱-荧光检测器(HPLC-FLD)结合,对4种阿非霉素建立了具有低检出限(LODs, 0.47~0.83 ng/g)和定量限(LOQs, 1.88~2.83 ng/g)的分析方法,并用于水稻和小麦中阿非霉素的检测,获得了良好的加标回收率(70.61%~113.30%)。为了进一步提高石墨烯的吸附性能和改善检测灵敏度,基于三维石墨烯的萃取材料被用于萃取环境水样中的三氯生,进而通过高效液相色谱-紫外检测(HPLC-UV),建立了相应的分析方法(LOD为0.070 mg/L; LOQ为0.22 mg/L),并成功应用于地表水中痕量三氯生的检测,加标回收率介于66%~72%之间^[7]^。

Hou等^[8]^将聚二烯丙基二甲基氯化铵(PDDA)修饰的氧化石墨烯接枝到SiO_2_上制备了一种固相萃取材料,通过静电相互作用对4种酸性除草剂表现出较强的萃取性能,建立了分析方法,给出了宽的线性范围(2.5~200 μg/L)和低的LODs(0.75~1.5 μg/L),适用于检测菠菜和葱等蔬菜中的除草剂残留。此外,具有强疏水性和良好选择性的*β*-环糊精(*β*-CD)也被用于改性氧化石墨烯,发展了*β*-CD@GO/SiO_2_萃取材料^[9]^,将柱固相萃取与高效液相色谱-二极管阵列检测器联用(SPE-HPLC-DAD),针对多种多环芳烃(PAHs)发展了一个具有宽线性范围(0.3~100 μg/L)和低LODs(0.1~0.3 μg/L)的分析方法,成功用于检测油炸食品中痕量的多环芳烃(PAHs)。基于不锈钢网(stainless steel mesh, SSM)表面可涂覆大量吸附剂材料、在萃取过程中可以改善传质速率的优点,Amiri等^[10]^利用溶胶-凝胶技术将氧化石墨烯/聚二甲基硅氧烷(GO/PDMS)修饰到圆盘状不锈钢网上。如图1所示,将其装进固相萃取柱管中发展了固相萃取与气相色谱-质谱联用(SPE-GC-MS)方法,对实际水样中的PAHs进行了灵敏检测。该萃取装置结合了不锈钢网(特征渗透率)、溶胶-凝胶涂层(高孔隙率、高比表面积和化学稳定性)和氧化石墨烯(高比表面积、化学稳定性以及强*π-π*作用)三者之间的优点,使得本方法表现出高灵敏度(0.2~1.0 pg/mL)和高富集倍数(2227~2367),实现了高灵敏度检测环境水样中PAHs。

**图1:**
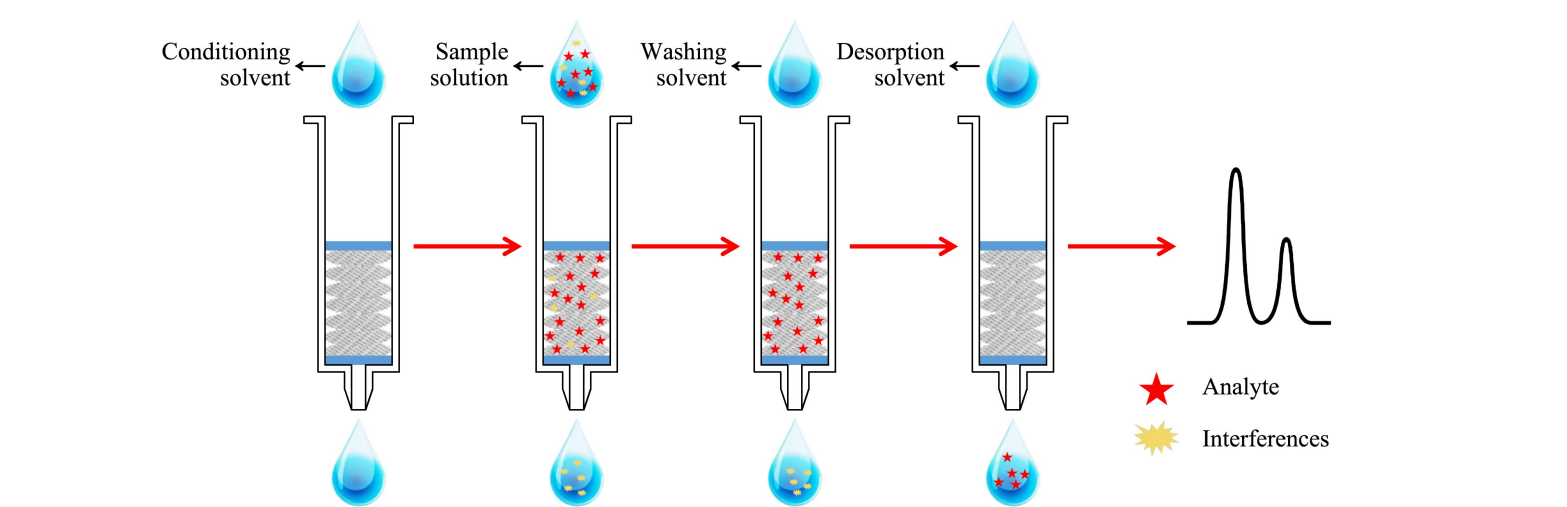
不锈钢网装填固相萃取柱的萃取过程示意图^[10]^

石墨烯气凝胶(GA)是一种由石墨烯构成的气凝胶材料,是世界上密度最小的固体材料,内部孔隙丰富且能够快速传质,其三维结构的特点也可以很好地避免石墨烯片层的聚集,有效提高了石墨烯与分析物的接触面积,从而改善萃取性能^[11,12]^。但GA较差的机械性能及单一的萃取选择性制约着其应用,为此,Gao等^[13]^将壳聚糖修饰到GA上,借助于壳聚糖的强静电和氢键相互作用增强GA机械性能,合成了石墨烯/壳聚糖复合气凝胶,表现出大的比表面积(440 m^2^/g)和蜂窝状的结构,能够提供氢键、*π-π*等相互作用。将该材料制备成固相萃取柱并联用GC-MS分析检测PAHs,实验结果证明分析方法的灵敏度高(1.7~8.8 pg/mL)、线性范围宽(10~2000 pg/mL)。Hou等^[14]^用PDDA对氧化石墨烯气凝胶(GOA)进行改性并修饰到机械性能强的不锈钢网上,然后将ZrO_2_纳米粒子沉积在表面,获得ZrO_2_/PDDA-GOA-SSM,将该材料应用于柱固相萃取中,富集蔬菜中的有机磷农药,建立了高灵敏度(LODs为0.2~1.0 μg/L)和宽线性范围(1.0~200 μg/L)的分析方法,并成功应用于检测蔬菜中的农药残留。此外,具有优异吸附性能、高比表面积、规整孔结构和良好化学稳定性的MOFs也被用于改性GA,制备了MIL-101(Cr)@GA复合萃取材料^[15]^,基于*π-π*、静电、氢键作用等多重萃取机理,发展了一种固相萃取-超高效液相色谱-串联质谱(SPE-UPLC-MS/MS)分析方法,成功检测了环境水样中的5种非甾体抗炎药,具有低的LODs(0.006~0.012 ng/mL)和良好的线性范围(0.05~5 ng/mL)。

常用的离线固相萃取方法存在耗时、耗力、误差大、样品通量低等缺点。针对这些问题,在线固相萃取技术被发展,它具有自动化和高通量的显著优势,在环境、食品、生物、医药等领域受到越来越多的关注。固相萃取装置与色谱检测仪器的在线联用,可实现样品制备和色谱检测的自动化,不仅显著减少了成本、分析时间和技术人员接触危险试剂的时间,而且改善了系统误差和检测准确性^[16,17]^。在线SPE-HPLC分析过程如图2所示,当六通阀处于Load状态时,样品溶液在样品泵的输送下流经固相萃取柱,目标分析物被萃取;萃取结束后,将六通阀转换为Inject状态,色谱流动相流经固相萃取柱将分析物洗脱下来进入液相色谱柱进行分离,最终进入检测器完成检测^[18]^。在固相萃取柱中直接装填纳米尺寸的石墨烯材料,会造成高柱压甚至柱堵塞,而将石墨烯嵌入通透性良好的整体柱中可以避免这个问题。Cui等^[19]^通过简单快速的自由基聚合方法,分别以1-辛烯(C8)、三甘醇二甲基丙烯酸酯(TEGDA)和1-十二醇/1-丙醇为单体、交联剂和致孔剂,以过氧化二苯甲酰-*N*,*N*-二甲基苯胺为引发剂,聚乙烯吡咯烷酮在氧化石墨烯片层间起支撑作用,以避免团聚并增强氧化石墨烯的分散效应,制备了一种氧化石墨烯杂化的聚合物整体柱(Poly(GO-co-C8-co-TEGDA))。将该整体柱作为在线固相萃取柱与液相色谱联用,成功应用于*β*-谷甾醇的在线富集和检测。此外,Chen等^[20]^利用氧化石墨烯和单一交联剂在不锈钢柱中发生原位聚合制备出了氧化石墨烯/聚乙二醇二甲基丙烯酸酯整体柱,与纯聚合物整体柱相比,它具有结构均匀、比表面积大等优点。将该整体柱作为固相萃取柱与HPLC-MS/MS在线联用,发展的在线分析方法能够在23 min内完成一个样品的分析,对牛奶样品和鸡肉样品中磺胺类药物的检测范围分别为1~100 μg/kg和2~200 μg/kg,说明该方法能够从食品样品中自动、快速和经济地富集和检测药物残留。Yang课题组^[21]^将氧化石墨烯键合到氨基化球形二氧化硅上制备出SiO_2_@GO核壳材料,被用于在线固相萃取富集无机汞和有机汞,以此来增强高效液相色谱-电感耦合等离子体质谱(HPLC-ICP-MS)的分析性能。该在线分析方法实现了良好的重现性(<5.0%)和高富集效应(1794~1963),可用于各种淡水资源中痕量汞污染物的检测,且富集和分离时间较短(<5 min),产生有毒废物较少,符合绿色化学的发展趋势。

**图2:**
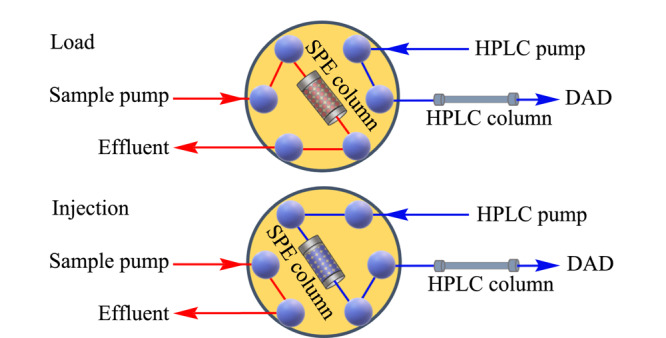
在线固相萃取-高效液相色谱分析过程示意图^[18]^

具有离域*π*电子结构的石墨烯材料作为一种性能优异的萃取材料,有着机械强度高、比表面积大、化学和热稳定性好等优点,在样品前处理领域中有着广泛的应用前景,但在生物样品中蛋白质的选择性富集方面的应用有待进一步研究。石墨烯的功能化能够满足特定的分析需求,将仍是样品前处理领域的研究热点。

## 2 分散固相萃取

利用*π-π*作用机理,石墨烯纳米片被应用于分散固相萃取水样中汞的有机络合物,结合原子吸收光谱(AAS)进行定量检测,发展了一个灵敏度高(LOD, 0.38 ng/L)和线性范围宽(0.38~1038 ng/L)的分析方法^[22]^。氧化石墨烯被应用于分散固相萃取稀土金属离子,借助于ICP-MS测定,发展了可以同时测定多种稀土金属的分析方法^[23]^。由于本研究所制备的氧化石墨烯对稀土金属表现出的吸附容量(6.1~12.2 mg/g)相似于或高于其他吸附材料,所以发展的分析方法能够获得满意的灵敏度,LODs和LOQs范围分别为0.03~1.08 ng/L和0.09~3.26 ng/L,成功应用于饮用水和坚果样品的分析。为了更加方便地利用氧化石墨烯进行分散固相萃取实验,利用聚电解质将氧化石墨烯层层组装到聚苯乙烯微球表面,发展了一种聚苯乙烯@氧化石墨烯(PS@GO)核壳型萃取材料,应用于分散固相萃取环境水样中的4种双酚类内分泌干扰物(双酚A、双酚B、双酚AF、四溴双酚A)^[24]^,在优化的条件下获得了73~103的富集倍数。联用HPLC-MS检测,构建了线性范围为0.1~1000 μg/L和LODs范围为0.02~0.11 μg/L的分析方法,成功应用于环境水样的分析检测。

为了克服氧化石墨烯自身的团聚,将二硫化钼嵌入氧化石墨烯片层之间,通过水热法制备了二硫化钼/氧化石墨烯复合材料(MoS_2_/GO),基于氢键和静电作用机理,应用于分散固相萃取4个对羟基苯甲酸酯类防腐剂(对羟基苯甲酸甲酯、乙酯、丙酯和丁酯),萃取时间仅为10 min且洗脱时间仅为1 min^[25]^。萃取后再通过超高效液相色谱检测,构建了分析方法,LODs和LOQs范围分别为0.4~2.3 ng/mL和1.4~7.6 ng/mL,成功应用于化妆品中对羟基苯甲酸酯防腐剂的检测,获得了满意的加标回收率(91.3%~124%)。其他研究人员也合成了该复合材料,通过超声辅助分散固相萃取方式,富集水中的重金属铬(VI)污染物,展示了583.5 mg/g的高吸附容量和3350倍的高富集效应,从而利用X射线荧光光谱给出了高检测灵敏度(LOD, 0.050 ng/mL),适用于环境水样中痕量铬的检测^[26]^。

高比表面积的氧化石墨烯也被用作基体材料,先在其表面原位生成镁铝双金属氢氧化物,然后再通过原位聚合反应生成磺化聚苯胺,制得氧化石墨烯/双金属氢氧化物@磺化聚苯胺复合材料(GO/LDH@SPAN)^[27]^。将其应用于超声辅助分散固相萃取4种邻苯二甲酸酯类塑化剂(邻苯二甲酸二甲酯、二丁酯、苯基丁基酯、二(2-乙基)己酯),借助于GC-MS发展了分析方法,LODs范围为0.06~0.3 ng/mL,成功应用于饮用水和蒸馏草药饮料中痕量塑化剂污染物的检测。氧化锌纳米颗粒被组装到还原氧化石墨烯上发展了一种复合材料,作为分散固相萃取材料,应用于包括黄曲霉毒素B1、B2、G1、G2等12种霉菌毒素的富集^[28]^。构建了DSPE-UPLC-MS分析方法,获得的LODs范围为0.03~0.14 μg/kg,应用于黄连样品中霉菌毒素的检测,得到了可信的实验结果。

上述研究表明石墨烯和氧化石墨烯是良好的分散固相萃取材料,可以通过功能化修饰来克服其团聚和进一步改善其萃取性能,适用于多种类型分析物的高效捕获。种类丰富的功能化材料以及多样性的功能化方式使得石墨烯在分散固相萃取技术领域中还有非常广阔的发展空间。

## 3 磁性固相萃取

磁性固相萃取材料通常由磁性纳米颗粒和具有良好萃取性能的功能化材料结合而成。磁性纳米颗粒是铁、钴、镍及其氧化物,它们具有良好的铁磁性或超顺磁性。Fe_3_O_4_纳米颗粒因制备简单、成本低、比表面积大和生物相容性好而常被用于磁性固相萃取领域。Fe_3_O_4_与萃取材料的复合方式主要分为两种:共沉淀法和化学修饰法。在合成磁性石墨烯材料时,共沉淀法是先合成石墨烯材料,然后在石墨烯表面原位生成Fe_3_O_4_纳米颗粒。如图3所示,Lu等^[29]^利用共沉淀法制备了磁性氧化石墨烯,并应用于精神药物的萃取,然后与UPLC-MS/MS联用,获得的LODs为0.02~0.2 μg/L。将方法应用于尿样中8种精神药物的分析,得到了加标回收率为80.4%~105.5%的可观结果。该方法为从尿液中萃取药物提供了一种方便、快速、绿色的样品前处理方法,可用于8种药物的同时高灵敏检测,具有较高的临床和法医学应用价值。该磁性萃取材料也被应用于食品分析领域,高效富集了多种姜制品中的姜酚,随后通过HPLC-MS/MS进行检测,开发了一种具有良好应用潜力的分析方法,可以用于姜产品的质量控制^[30]^。在环境中重金属污染控制方面,该萃取材料能够快速富集水中的铊(Ⅰ)和铊(Ⅲ)^[31]^,与AAS相结合,发展了检测痕量铊的分析方法。以上工作充分证明了磁性氧化石墨烯具有良好的萃取行为和应用潜力,基于其建立的分析方法可适用于多种目标分析物的检测。

**图3:**
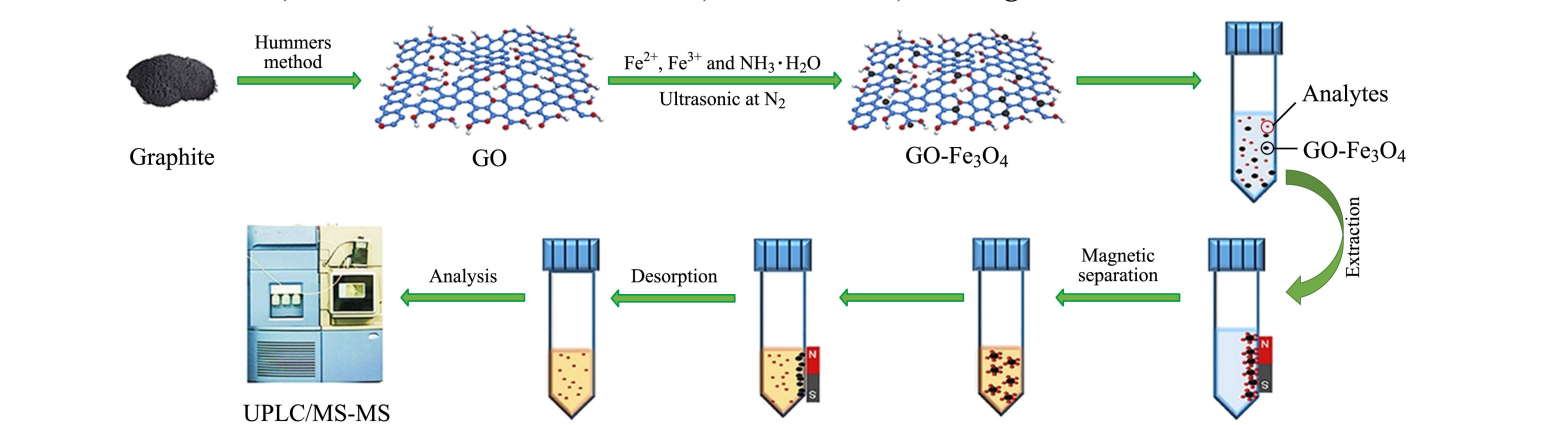
共沉淀法制备磁性氧化石墨烯萃取材料及应用于磁性固相萃取过程示意图^[29]^

上述研究工作中氧化石墨烯与Fe_3_O_4_通过物理吸附的方式进行结合,存在磁性颗粒易脱落的缺点。共价键合法能够克服这个问题。通常为先合成Fe_3_O_4_纳米球,然后利用正硅酸乙酯包覆一层SiO_2_,再利用3-氨基丙基三乙氧基硅烷(APTES)进行氨基化,最后在1-乙基-3-(3-二甲基氨基丙基)碳二亚胺盐酸盐(EDC)和*N*-羟基琥珀酰亚胺(NHS)存在的条件下进行脱水缩合反应实现与氧化石墨烯成功结合(Fe_3_O_4_@GO)(见图4)^[32]^。Medina等^[32]^将上述材料用于萃取水样品中的5种二苯甲酮,基于疏水相互作用机理,并借助于纳米颗粒的磁性和氧化石墨烯的优良吸附能力,表现出良好的萃取行为。萃取完后采用HPLC-MS/MS进行定量分析,获得的LODs低至2.5 μg/L,分析重现性良好(RSD, 1.3%~9.8%)。

**图 4:**
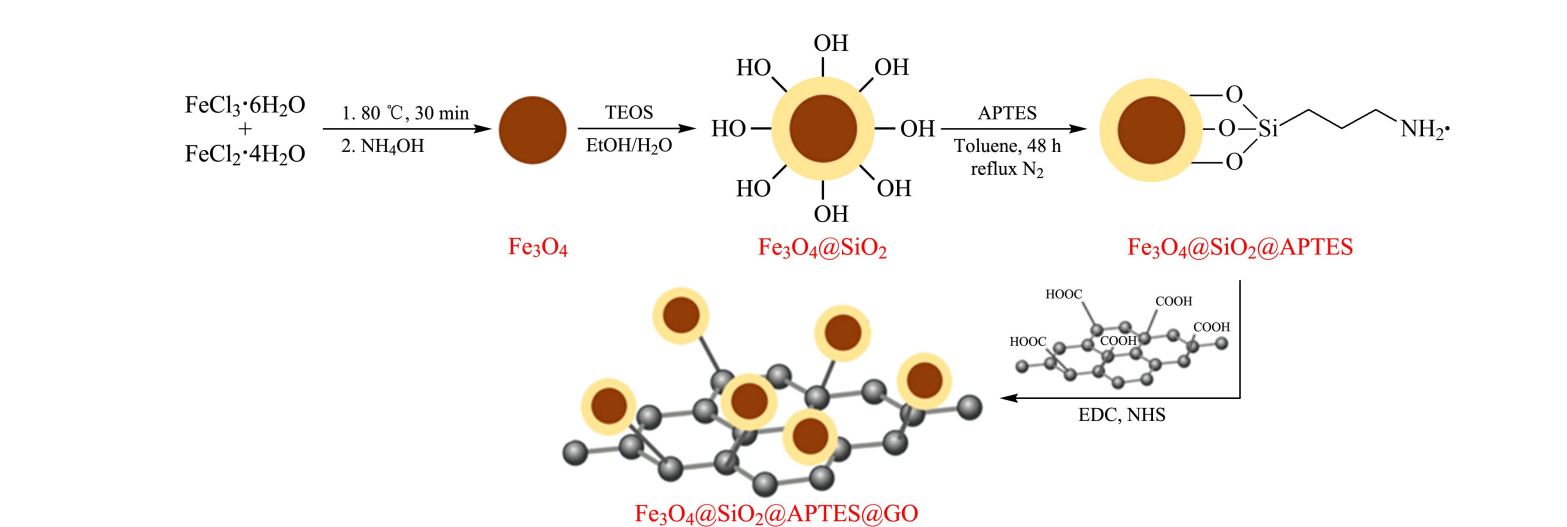
化学修饰法制备磁性氧化石墨烯萃取材料过程示意图^[32]^

将建立的分析方法应用于游泳池水样的分析,获得了令人满意的加标回收率(86%~105%)。Yang等^[33]^利用此萃取材料实现了水中剧毒污染物砷(V)的萃取,结合石墨炉原子吸收光谱(GF-AAS)检测,建立了灵敏的分析方法,LOD为1.02 ng/L,富集倍数为392,应用于环境水和饮用水中(超)痕量砷(V)的检测,结果令人满意。因此,基于该材料所构建的分析方法为快速检测水样中的痕量污染物提供了可行方案。

然而,氧化石墨烯的萃取选择性单一,对其进行改性是提高萃取选择性的关键,可以大大拓展氧化石墨烯的应用范围。在磁性氧化石墨烯的基础上,通过进一步改性可以获得高选择性的磁性固相萃取材料。比如,Mustafa课题组^[34]^制备了邻苯二酚紫浸渍的Fe_3_O_4_-氧化石墨烯材料,基于邻苯二酚紫与Cu^2+^的配位机理,通过磁性固相萃取选择性富集了Cu^2+^,然后利用火焰原子吸收光谱(FAAS)测定,建立了良好的分析方法(LOD为4.0 μg/L, RSD为4.93%),成功应用于水、红茶和膳食补充剂中Cu^2+^的测定。然而,具有选择性萃取作用的官能团仅仅被物理吸附在氧化石墨烯表面,存在易脱落、稳定性差的问题,Wang课题组^[35]^通过化学键合法对石墨烯进行功能化可以有效改善这一缺陷。他们以氨基化磺胺二甲氧嗪为适配体,在EDC和NHS作为羧基活化剂和助缩剂的条件下,与磁性氧化石墨烯表面的-COOH缩合,将适配体成功接枝,获得功能化磁性石墨烯材料(Apt-Fe_3_O_4_-GO)。将该磁性固相萃取材料与HPLC联用,对牛奶中的磺胺二甲氧嘧啶实现了高选择性检测,加标回收率为75.9%~92.3%。

Xu等^[36]^通过水热法将氧化石墨烯、Fe_3_O_4_和MOF(UiO-67)前驱体进行复合,制备了Fe_3_O_4_@GO@UiO-67。与UiO-67@Fe_3_O_4_进行比较,结果说明氧化石墨烯的引入不仅不会改变UiO-67@Fe_3_O_4_纳米颗粒的球形结构,而且还可以减少颗粒的团聚。同时,UiO-67@Fe_3_O_4_颗粒分散在氧化石墨烯薄片的褶皱中,也减少了氧化石墨烯的团聚。以该材料为磁性固相萃取吸附剂,与UPLC-MS/MS联用,建立了一种快速且简便的分析方法,测定了蜂蜜中7种硝基咪唑和5种苯并咪唑兽药残留。本分析方法对12种兽药的LODs范围为0.2~0.6 μg/kg,在实际样品中的加标回收率为70.5%~103.4%。此工作不仅有效克服了石墨烯、UiO-67@Fe_3_O_4_的自身团聚问题,而且为复杂样品基质中的兽药残留检测提供了有效方法。与此类似,Chen等^[37]^也利用金属有机框架材料对氧化石墨烯进行功能化用于磁性固相萃取,与高效液相色谱联用建立了分析食品样品中环氧康唑农药的新方法。

氧化石墨烯应用于磁性萃取材料,主要提供*π-π*堆积、疏水、亲水作用等萃取机理,萃取选择性差,因此研究工作主要集中在功能化石墨烯萃取材料方面。氧化石墨烯可以与有机小分子、MOFs等进行有机结合,从而使得具有理想选择性、优异萃取效率的功能化磁性石墨烯萃取材料应用于食品、环境、生物等样品中农药、兽药、重金属离子、砷、精神类药物、有机污染物等的检测,得到了令人满意的效果。

## 4 搅拌棒固相萃取

Baltussen等^[38]^在1999年首次报道了搅拌棒吸附萃取技术,该技术具有操作简便、萃取效率高、环境友好等优点^[39]^。萃取涂层是搅拌棒固相萃取的关键部分,它决定了对目标分析物的选择性、萃取回收率以及萃取重现性。

我们课题组^[40]^通过简便的火焰沉积法在聚四氟乙烯磁子表面原位生成碳纳米颗粒涂层,制备了一种固相萃取搅拌棒,通过疏水作用萃取机理,可以对环境水中的PAHs进行高效富集,联用HPLC-DAD分析获得的LODs为3 ng/L。石墨烯作为一种先进的碳纳米材料也已经被应用于发展固相萃取搅拌棒,Aghaei等^[41]^制备了一种硫功能化的氧化石墨烯涂层搅拌棒,用于贵金属离子的快速富集。他们将异氰酸酯基改性的搅拌棒置于氧化石墨烯的分散液中,得到氧化石墨烯键合的搅拌棒,随后浸入含有双-(*γ*-三乙氧基硅基丙基)四硫化物的溶液中搅拌并回流,获得硫功能化的氧化石墨烯涂层。借助于石墨烯的高比表面积和强吸附能力以及硫和贵金属离子之间的配位作用,该搅拌棒可以从水溶液中高效富集钯(Ⅱ)、金(Ⅲ)和银(Ⅰ)。

此外,将性质不同的两种及两种以上的材料进行结合的复合材料能够提供更强的萃取性能。泡沫镍(NF)是一种多孔磁性金属材料,具有良好的机械强度、多孔结构和较大的比表面积^[42]^,三维骨架和粗糙的表面结构有利于增大与分析物的接触面积和提高传质效率。Hu课题组^[43]^通过水热法将氧化石墨烯还原并均匀负载在NF搅拌棒表面,获得G-NF搅拌棒,石墨烯涂层能够与苯并三唑类紫外线吸收剂产生*π-π*和疏水相互作用而被吸附。结合HPLC-DAD,对环境水中的6种紫外线吸收剂进行了检测,建立了线性范围和LODs分别为1~100 μg/L和0.33~0.50 μg/L的分析方法。与商用PDMS搅拌棒相比,本研究所发展的搅拌棒具有吸附和脱附速度更快、消耗有机溶剂更少等优点。降低萃取涂层厚度、减小搅拌棒直径并同时增加吸附作用位点,能够促进萃取过程中分析物的传质和提高搅拌棒固相萃取方法的萃取效率。Mohammadi等^[44]^将纺丝聚丙烯中空纤维包裹在搅拌棒外侧作为萃取涂层载体,制备了一种马来氨酸功能化的氧化石墨烯磁性吸附剂(Fe_3_O_4_@MFGO)。在表面活性剂TritonX-140的辅助作用下,借助于超声波将Fe_3_O_4_@MFGO固定在中空纤维的孔道中,中空纤维克服了生物样品基体的干扰,利于吸附剂与分析物的充分接触,可以提高萃取效率。MFGO表面的羟基和羧基与药物分析物相互作用,利于获得高选择性。利用制备的Fe_3_O_4_@MFGO@SBSE进行萃取,并结合GC-MS,对非甾体抗炎药和去肾上腺素抑制剂药物建立分析方法,该方法具有宽的线性范围(0.49~743 ng/mL)和低的LODs(0.12~0.17 ng/mL),实现了从人类尿液中检测阿司匹林、布洛芬和文拉法辛,加标回收率大于97.2%。

搅拌棒固相萃取方法的萃取时间比较长,会降低样品通量。分散固相萃取能改善这一缺点,它通过分散微/纳米萃取材料,使之与目标分析物进行充分接触,从而促进快速传质和缩短萃取平衡时间,提高萃取效率。Salvador课题组^[45]^将分散固相萃取和搅拌棒固相萃取相结合,设计了搅拌棒吸附分散微萃取,可以对目标分析物进行更快和更容易的萃取和脱附。为了快速萃取化妆品中的PAHs,该课题组将CoFe_2_O_4_嵌入还原氧化石墨烯(rGO)片层间制成磁性复合材料(CoFe_2_O_4_/rGO)作为搅拌棒吸附分散微萃取吸附剂涂层。rGO的高比表面积和大*π*共轭结构使其与PAHs之间形成*π-π*相互作用,显著增强了材料的萃取性能。本研究所发展的CoFe_2_O_4_/rGO@SBSDME-GC-MS方法的灵敏度高(LODs, 0.28~24.22 ng/g),对洗面奶、乳液等化妆品中10种PAHs的萃取速度快,只需要10.5 min就可以达到萃取平衡。

## 5 纤维固相微萃取

纤维固相微萃取作为固相微萃取的一种形式,已被广泛应用于环境监测、食品安全、药物分析等领域。开发具有优异萃取效率、良好选择性和稳定性的纤维萃取涂层一直是该领域的研究热点。

我们课题组^[46]^利用溶胶-凝胶法将氧化石墨烯逐层键合到预处理的不锈钢丝表面,然后通过水合肼将其还原为石墨烯,发展了石墨烯涂层键合的不锈钢丝固相微萃取纤维,通过疏水作用机理萃取水中的多种烷烃污染物,借助于气相色谱分析,获得的灵敏度达到0.05 μg/L;还利用离子液体作为桥链,将氧化石墨烯层层组装到不锈钢丝表面,发展了另一种石墨烯涂层的萃取纤维,与气相色谱联用,应用于分析检测环境水样中的多种PAHs^[47]^。近年来,石墨烯依然是具有吸引力的萃取纤维涂层材料,Yu等^[48]^将锌丝插入氧化石墨烯的水分散液中,使氧化石墨烯自组装在锌丝表面形成萃取涂层,将该萃取纤维与GC-MS联用检测环境水样中的7种多氯联苯,LODs为0.03~0.2 ng/L,线性范围为1~200 ng/L。该纤维不仅制备简单、环保,而且其萃取能力是商品化PDMS纤维的1.96~5.07倍,因此具有很大的优势。采用等离子体处理方法将铅笔芯表面的石墨原位氧化并剥离成多孔氧化石墨烯,发展了一种石墨烯涂层的原位制备方法^[49]^,该氧化石墨烯涂层的纤维在顶空固相微萃取模式下与GF-AAS联用,对于水样中镉离子的LOD为5 ng/L,线性范围为0.04~0.26 μg/L,萃取重现性的RSD为2.1%。与商品化萃取纤维和其他纤维不同,该新型纤维的制备环保、低成本、无毒,且由于是原位制备的萃取涂层,不存在涂层脱落的问题。此外,纤维表面含有丰富的含氧官能团,对镉表现出较高的萃取效率,成功用于测定自来水、河水和池塘水中的镉。Kazemi等^[50]^另辟蹊径,将多壁碳纳米管经水热处理得到氧化石墨烯纳米带(GONRs),呈现部分展开形态,使整个结构疏松多孔,有效避免了氧化石墨烯团聚导致的比表面积大幅度下降的问题。合成的GONRs作为纤维的萃取涂层从水中顶空萃取6种邻苯二甲酸酯塑化剂,采用GC-FID进行检测,LODs为0.02~0.2 μg/L。与多壁碳纳米管涂层纤维和商用PDMS纤维相比,本研究所发展的萃取纤维表现出更优异的萃取能力。

此外,将石墨烯与其他材料进行掺杂或修饰制成复合材料,不仅能有效改善石墨烯的团聚,而且能选择功能化材料来调控萃取性能。Beiranvand等^[51]^将一种有机硅烷功能化氧化石墨烯固定到铂丝表面,制备了3-氨丙基三乙氧基硅烷交联氧化石墨烯(GO/APTES)复合固相微萃取涂层,并采用超声波辅助顶空萃取与GC-FID联用,对土壤中苯系物(苯、甲苯、乙苯、二甲苯)进行检测,在最佳萃取条件下,LODs为0.1~0.4 ng/g,工作曲线在2.4~5000 ng/g内呈线性关系。Sereshti等^[52]^制备了氧化石墨烯/聚苯胺(GO/PANI)导电纳米复合材料,将其涂覆在铜丝上作为萃取纤维,采用电场增强萃取与HPLC-UV联用,对水和牛奶样品中土霉素、四环素和强力霉素3种抗生素进行检测,分别获得了对应的LODs为0.32~1.01和2.42~7.59 μg/L,并成功应用于实际样品分析。为解决纤维固相微萃取涂层易脱落的问题,Hajebi等^[53]^先制备了聚酰胺/氧化石墨烯/聚吡咯(PAM/GO/PPY)复合材料,再采用静电纺丝技术将其牢牢地固定在不锈钢丝表面,制得一种具有稳定萃取涂层的纤维,通过顶空萃取富集水样中甲基苯丙胺,联用GC-MS对目标分析物进行检测,LOD可低至0.9 μg/L,日内和日间重现性RSD(*n*=3)均小于9.7%,纤维间重现性的RSD(*n*=3)小于12.3%。Du等^[54]^以MnO_2_修饰的6-氨基己酸功能化石墨烯(MnO_2_-fGr)与1-乙基-3-乙烯基咪唑双(三氟甲基磺酰基)酰亚胺([AVIm]NTf_2_)作为原料,在不锈钢丝上电沉积制成一种新型的聚吡咯复合涂层材料(Ppy/MnO_2_-fGr/[AVIm]NTf_2_),并将其作为萃取纤维,以4种苯甲酸酯(苯甲酸甲酯、乙酯、丙酯、丁酯)作为分析物,与GC-FID联用,在最佳条件下,富集倍数为140~460,线性范围为25~60000 ng/L, LODs为2.84~6.42 ng/L。将该方法应用于化妆品中苯甲酸酯的检测,其加标回收率为82.8%~116.8%。Li等^[55]^合成了一种新型六方氮化硼(BN)改性rGO材料,并将其作为萃取材料涂覆在不锈钢丝上,用于萃取水和土壤样品中的7种PAHs,与GC-FID联用,分析水样的LODs为0.05~0.15 μg/L,分析土壤样品的LODs为0.3~0.5 ng/g,此外还进行了对比试验,该萃取纤维萃取能力远超rGO或者BN涂覆的萃取纤维。

共价有机框架材料(COFs)和MOFs具有较大的比表面积、可调控的孔径等优点,可以与石墨烯结合制成理想的萃取材料。Khataei等^[56]^将氧化石墨烯键合到共价有机框架材料表面,用于提高其疏水性,制成rGO/席夫碱网络-1(SNW-1)复合材料,使用聚醚砜作为黏附剂修饰在不锈钢丝表面制成萃取纤维,这种拥有稳定涂层的纤维在顶空萃取模式与GC-MS联用,检测水样中的6种邻苯二甲酸酯塑化剂,LODs为0.01~0.50 μg/L,纤维的萃取重现性的RSD低至6.8%,制备重现性低至9.2%。将该方法用于实际样品的分析,回收率为80.5%~111.0%。随后,Gao等^[57]^在APTES改性的氧化石墨烯中加入不同比例的共价有机框架材料单体(1,3,5-三甲酰基间苯三酚(Tp)和联苯胺(BD)),制备了一系列的TpBD/GO复合材料,随单体浓度的逐渐增加,该涂层材料的萃取能力先增加后减弱,主要是由于氧化石墨烯表面的位点会被高负载量的COFs所掩盖。将最佳性能的萃取纤维与恒流解吸电离质谱(CFDI-MS)联用,对于双酚A的LOD低至22.2 ng/L,同时,该纤维的萃取效率分别是TpBD涂层纤维和氧化石墨烯涂层纤维的2.2倍和4.7倍,分析时间也大大缩短。利用相似于上述在氧化石墨烯表面原位生成复合材料的制备方式,Liu等^[58]^用水热法发展了一种GO/Zr-MOF材料,并将其作为纤维萃取涂层用于水中布洛芬和双氯酚酸钠盐的富集,与GC-FID联用获得的LODs可达0.001~0.030 μg/L,线性范围宽至0.01~500 μg/L。与氧化石墨烯涂层纤维和Zr-MOF涂层纤维相比,该纤维在灵敏度和线性范围等多个方面均有优势。Khodayari等^[59]^将石墨烯纳米粉与金属有机框架(MIL-53(Al))进行掺杂,用作萃取纤维的涂层并与GC-MS联用,对食品和环境水样中4种有机磷农药的LODs可达0.2~1.5 ng/g,线性范围为0.8~600 ng/g。该方法重现性好,RSD为4.5%~7.3%,回收率为88%~109%。

气凝胶材料具有高孔隙率、高比表面积的特性,所以其适合作为萃取材料应用于样品前处理领域,Peng等^[60]^将氧化石墨烯引入到壳聚糖气凝胶(CS)中,制备了一种稳定的GO/CS复合材料,作为涂层制得萃取纤维,与GC-MS联用,对环境水样中的5种PAHs进行检测;在最佳萃取条件下,多环芳烃的线性范围宽(0.5~1000 ng/L),富集倍数高(311~3740), LODs低(0.03~1.28 ng/L),加标回收率满意(91.6%~110%)。Wang等^[61]^采用物质的量比为2:1的葡萄糖和氯化胆碱组成的低共熔溶剂对氧化石墨烯进行改性,制备了一种石墨烯基气凝胶涂层萃取纤维,采用顶空固相微萃取,与GC-MS/MS联用,将该方法成功应用于虾类中多氯萘的检测,加标回收率为80.4%~108%, LODs可以低至0.00983 pg/g。值得一提的是,从萃取纤维制作到分析检测过程,该研究几乎未使用有机溶剂,是一种非常环保的分析技术。

综上所述,石墨烯材料不仅因疏水性、*π-π*作用等对疏水性物质具有萃取能力,而且氧化石墨烯的表面官能基团赋予其理想的修饰潜力。针对石墨烯作为固相微萃取涂层易团聚、易脱落等问题,科研工作者们利用功能化的方法,来改善石墨烯涂层材料的萃取性能。

## 6 管内固相微萃取

20世纪末,一种可与HPLC进行在线联用的样品前处理技术——管内固相微萃取被首次引入。管内固相微萃取与HPLC的在线联用是将萃取管连接到六通阀上^[62]^,如图5所示,通过转动六通阀实现样品溶液的萃取和洗脱过程。最早选用毛细管气相色谱柱作为萃取管,后来发展了多种类型的萃取管,包括内壁涂覆型^[63,64]^、整体柱型^[65,66]^、颗粒填充型以及纤维填充型萃取管^[67]^等。由于萃取涂层材料的性能决定着萃取行为,因此,开发高效的萃取涂层是管内固相微萃取的研究核心。近年来,石墨烯和氧化石墨烯由于其优异的萃取性能,也被作为萃取涂层材料引入管内固相微萃取研究领域^[65⇓⇓⇓-69]^。

**图5:**
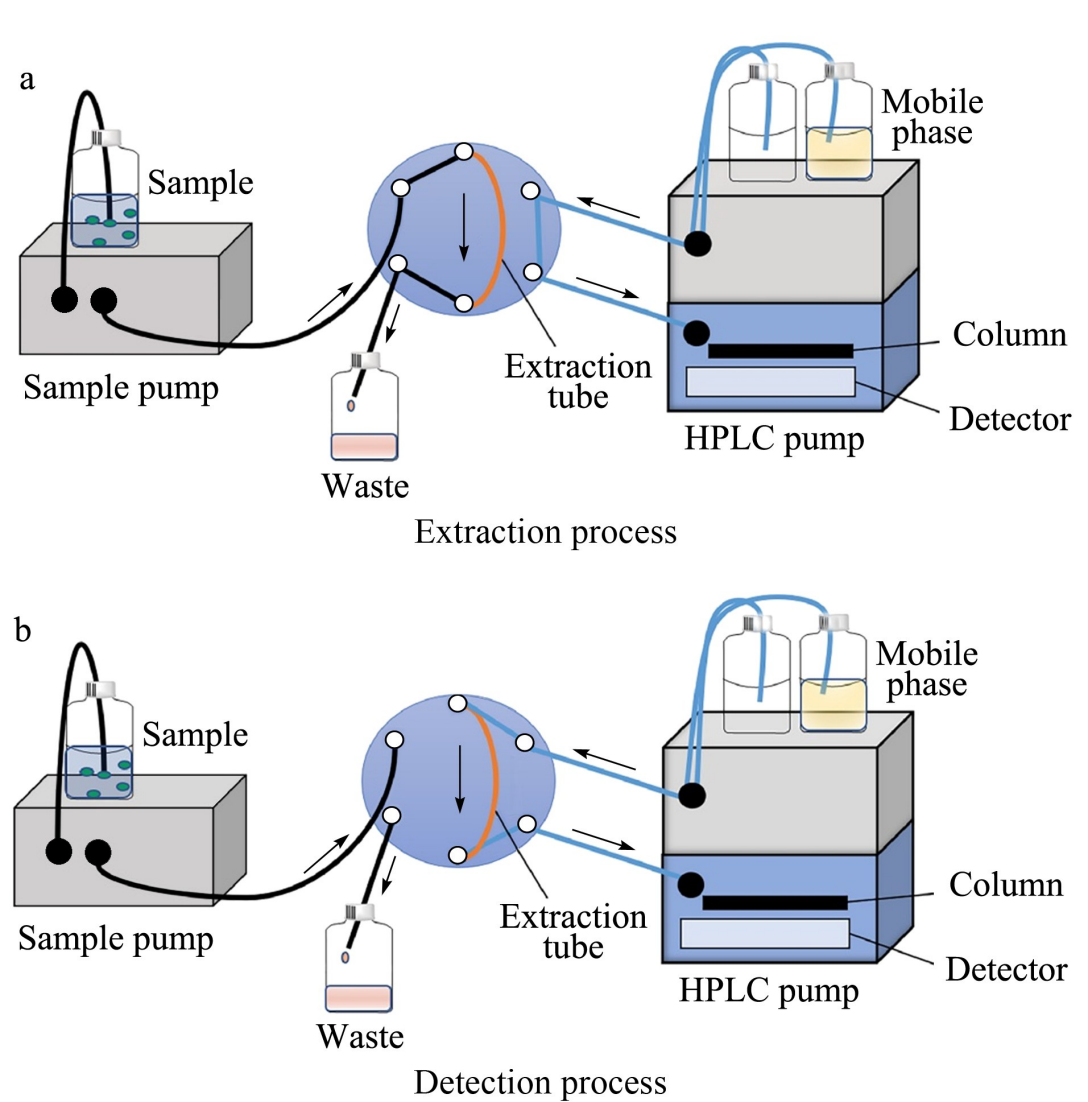
管内固相微萃取-高效液相色谱在线联用分析示意图^[62]^

借助电化学沉积法将石墨烯修饰到不锈钢管内壁上,已经发展了多种内壁涂覆型萃取管。利用3,4-二氧噻吩(EDOT)和吡咯(Py)作为单体,通过电化学方法在不锈钢管内壁沉积形成氧化石墨烯杂化聚(噻吩-吡咯)(GO/PEDOT/PPy)涂层^[68]^,建立了在线管内固相微萃取-高效液相色谱-质谱(IT-SPME-HPLC-MS)方法,用于检测尿液中8-羟基脱氧鸟苷、3-羟基菲和1-羟基芘,该方法具有较好的灵敏度(4~41 pg/mL)和良好的加标回收率(71.6%~109.5%)。实验结果表明该材料的稳定性良好、萃取效率高、抗基体干扰性强,所发展的分析方法具有成本低、操作简便、灵敏度高、快速、自动化等优点,实现了在线检测多种PAHs。Seidi等^[69]^将离子液体功能化氧化石墨烯(GO-IL)沉积到不锈钢管内表面形成萃取涂层,利用该萃取管对蜂蜜样品中的萘进行管内循环顶空固相微萃取(CHS-IT-SPME),进而在线联用HPLC分析。所发展的分析方法获得了低的LOD(0.1 ng/mL)和LOQ(0.3 ng/mL),并提供了较宽的线性范围(0.3~200 ng/mL)和满意的加标回收率(90.0%~106.5%)。本研究所提出的顶空管内固相微萃取可以避免样品直接流经萃取管对涂层的破坏,其作为一种独特的萃取方式为管内固相微萃取的拓展应用提出了一个新思路。

针对内壁涂覆型萃取管萃取容量小、死体积大等缺点,发展了整体柱型萃取管。整体柱具有双连续结构和双孔分布、良好的渗透性、优良的萃取能力、快速洗脱等优点,能够实现高通量分析。Zhao等^[70]^发展了一种氧化石墨烯功能化的有机-无机杂化整体柱萃取管,先利用甲基丙烯酰氧乙基三甲基氯化铵(META)对氧化石墨烯进行修饰,进而与二氧化钛纳米颗粒结合,最后将复合物(GO-META/TiO_2_)引入有机杂化整体柱反应体系中制得整体柱。将基于该整体柱的管内固相微萃取与基质辅助激光解吸电离飞行时间质谱(MALDI-TOF-MS)联用构建了分析方法,用于复杂生物样品中磷酸化肽的检测。结果表明,氧化石墨烯功能化的整体柱对生物样品中的磷酸化肽给出了高选择性和良好富集能力。基于上述研究,该课题组进一步采用原位紫外光聚合法制备了另一种石墨烯掺杂多孔聚合物整体柱作为固相微萃取管^[71]^,通过光聚合反应得到聚合物整体柱,建立了管内固相微萃取-毛细管电泳-激光诱导荧光法(IT-SPME-CE-LIF)测定磺胺类药物的分析方法,给出了宽的线性范围(2~500 μg/L)、低的LODs(0.25~0.47 μg/L)以及满意的加标回收率(91.1%~94.6%),该研究不仅成功应用于检测牛奶中磺胺药物残留,而且为其他食品基质中磺胺类药物的检测提供了思路。

石墨烯还被包覆到球形载体上应用于填充型固相微萃取管,Mejía-Carmona等^[72]^用氧化石墨烯包覆氨丙基修饰的球形二氧化硅,然后再分别经过氢化还原、三甲基氯硅烷修饰、十八烷基硅烷修饰制备得到3种功能化石墨烯材料,将这些材料填充到固相微萃取管中,与超高效液相色谱-串联质谱联用,建立在线分析方法,并以黄嘌呤为模型分析物评价这些材料的萃取性能,实验证明,十八烷基硅烷功能化石墨烯(SiGOC_18_ecap)萃取黄嘌呤的性能优于另外两种材料,得到了较为满意的结果:LOQs介于0.3~1.0 μg/L之间,RSD小于10%,并成功用于烘焙咖啡样品中黄嘌呤的检测。这项工作利用载体发展核壳型功能化石墨烯材料,是一种良好的设计思路,拓展了石墨烯在管内固相微萃取领域的应用潜力。

我们课题组^[73]^发展了多种纤维填充型萃取管,也将氧化石墨烯电沉积到碳纤维表面,然后把碳纤维束装填到聚醚醚酮管中发展了一种新型的石墨烯涂层纤维填充型萃取管,与HPLC-DAD联用,针对污水中8种PAHs构建了在线富集分析方法,富集倍数高达3000倍,检出限低至1 ng/L。氧化石墨烯的超高机械强度能够弥补有机气凝胶干燥过程中体积收缩、孔道坍塌、孔隙率下降、比表面积减小等缺点,而且还能增加吸附位点。我们课题组^[74]^将氧化石墨烯引入有机气凝胶中,发展了一种氧化石墨烯功能化三聚氰胺-甲醛气凝胶(GO-MF),将气凝胶粉末涂覆到不锈钢丝上,装进一根聚醚醚酮管内得到纤维填充型固相微萃取管。将其与HPLC-DAD联用,对多种PAHs进行分析,检测灵敏度达到1 ng/L,线性范围在0.003~20.0 μg/L之间,成功应用于水中多环芳烃的检测,实验证明该萃取管具有良好的使用寿命和化学稳定性。

综上所述,石墨烯和氧化石墨烯作为萃取材料已经被广泛应用于管内固相微萃取,所发展的IT-SPME-HPLC在线分析方法已成功应用于多类别分析物,比如PAHs、抗生素、生物分子、药物残留等的高效分析检测。

## 7 结论

本文总结了自2020年至今石墨烯萃取材料在样品前处理领域的最新研究进展,讨论了石墨烯、氧化石墨烯以及各种材料(有机基团、离子液体、分子印迹聚合物、纳米材料、磁性材料、金属有机框架、共价有机框架等)功能化石墨烯吸附剂,在柱固相萃取、分散固相萃取、磁性固相萃取、搅拌棒萃取、纤维固相微萃取、管内固相微萃取等主要的样品前处理技术中的具体应用,高效萃取和选择性富集了多种类型的分析物,如金属离子、有机污染物、药物分子、农药残留、兽药残留等,在环境、食品、医学等诸多领域具有广泛的应用(具体见附表,www.chrom-China.com)。石墨烯存在易团聚和吸附选择性差等问题,这制约着它在样品前处理领域的进一步应用,而氧化石墨烯含有丰富的官能团,易于功能化,通过对氧化石墨烯进行功能化成功拓展了石墨烯材料的应用空间。未来石墨烯在样品前处理领域的发展主要趋向于以下几个方面:1)通过专一选择性的基团对石墨烯进行功能化,如亲和基团、螯合基团、分子印迹位点等,赋予石墨烯材料独特的萃取选择性;2)利用具有优异特性的先进材料如共价有机框架、金属有机框架、气凝胶、纳米材料等与石墨烯相结合,一是在石墨烯基体表面原位生成功能化材料,二是利用石墨烯修饰和改性这些基体材料,实现材料间的优势互补,发展高性能的石墨烯基杂化材料,在样品前处理领域具有广阔的应用前景;3)将电磁材料与石墨烯结合形成具有电磁特性的石墨烯复合材料,在样品前处理过程中借助电磁场辅助,提升萃取选择性和萃取效率,并且方便回收和快捷分离萃取材料;4)复杂样品的前处理一直是比较困难的,石墨烯基材料在复杂样品前处理中展现了良好的应用前景,将来会被更多地应用于解决该问题;5)开发更多的绿色方法,制备石墨烯萃取材料或实现对石墨烯的功能化,更加符合绿色化学的发展趋势;6)应该更加注重发展石墨烯基在线样品前处理材料,比如在线固相萃取柱、固相微萃取管等,以便适应分析化学的重要发展方向之一的在线分析。
